# R3DMCS: a web server for visualizing structural variation in RNA motifs across experimental 3D structures from the same organism or across species

**DOI:** 10.1093/bioinformatics/btae682

**Published:** 2024-11-15

**Authors:** Sri Devan Appasamy, Craig L Zirbel

**Affiliations:** Protein Data Bank in Europe, European Molecular Biology Laboratory, European Bioinformatics Institute (EMBL-EBI), Wellcome Genome Campus, Hinxton, Cambridge CB10 1SD, United Kingdom; Department of Biological Sciences, Bowling Green State University, Bowling Green, OH 43403, United States; Department of Mathematics and Statistics, Bowling Green State University, Bowling Green, OH 43403, United States

## Abstract

**Motivation:**

The recent progress in RNA structure determination methods has resulted in a surge of newly solved RNA 3D structures. However, there is an absence of a user-friendly browser-based tool that can facilitate the comparison and visualization of RNA motifs across multiple 3D structures.

**Results:**

We introduce R3DMCS, a web server that allows users to compare selected RNA nucleotides across all 3D structures of a given molecule from a given species, or across all 3D structures mapped to a single Rfam family. Starting from one instance of the motif, R3DMCS retrieves, aligns, annotates, organizes, and displays 3D coordinates of corresponding sets of nucleotides from other 3D structures. With R3DMCS, one can explore conformational changes of motifs due to 3D structures being solved in different functional states or different experimental conditions. One can also investigate conservation of 3D structure across species, or changes in 3D structure due to changes in sequence.

**Availability and implementation:**

R3DMCS is open-source software and freely available at https://rna.bgsu.edu/correspondence/ and https://github.com/BGSU-RNA/RNA-3D-correspondence.

## 1 Introduction

Advances in X-ray crystallography and cryo-EM experimental techniques coupled with the interest generated by the abundance of functional RNA molecules have led to an expansion of the number and variety of RNA structures being determined (Lawson *et al.* 2024) (https://www.nakb.org/statistics.html). We have many structures of the same molecule from the same organism (e.g. over 500 *Thermus thermophilus* ribosomal small subunit structures) and we have many structures of the same molecule from different organisms (e.g. over 600 ribosomal large subunit structures from over 30 organisms). As a result, knowledge regarding the molecular interactions that shape RNA motifs and architecture has greatly increased (Petrov *et al.* 2013, Chojnowski *et al.* 2014, Appasamy *et al.* 2016, Richardson *et al.* 2020).

RNA function often involves a series of conformational changes, which can be considered as different conformational states that can be adopted by the same RNA molecule (Ganser *et al.* 2019). These different conformational states collectively form an ensemble. A single RNA fold can represent the dominant structure in the ensemble when it is stabilized by a set of RNA 3D motifs and/or bound protein. In order to better understand RNA and RNA-mediated biological processes, it is vital to uncover the changes that occur when the structure transitions between the different conformations. These can range from complex motions involving domain movements to simple rearrangement of loop nucleotides. At the same time, different species sometimes have different sequences forming the same RNA motif, so that sequence changes but the 3D structure remains the same, or sometimes adopts a different geometry entirely. Both are of interest.

Given the sheer number of experimental structures of the same RNA molecule that have been solved under varying experimental conditions, there is an urgent need for an automated approach to compare RNA 3D motifs and their associated interactions in these structures. Here, we present a new web server called RNA 3D Motif Correspondence Server (R3DMCS) to aid the retrieval, visualization, and comparison of instances of RNA 3D motifs (having length up to around 30 nucleotides) across many 3D structures. Starting from a query instance of the motif, R3DMCS compares motif instances geometrically, organizes them according to their geometry, and makes it easy to superimpose and compare their 3D structures.

## 2 Materials and methods

Since 2011, the BGSU RNA group has maintained a data pipeline that downloads new RNA-containing 3D structures weekly, annotates them with pairwise interactions, extracts hairpin, internal, and 3-way junction motifs, groups together chains of the same molecule from the same organism into equivalence classes (EC), and selects representative structures from each equivalence class (Sarver *et al.* 2008, Leontis and Zirbel 2012, Parlea *et al.* 2016). The equivalence classes and their representatives can be seen on the RNA 3D Hub website at https://rna.bgsu.edu/rna3dhub/nrlist.

As part of the data annotation pipeline, we compute and store the correspondences between residues in each pair of chains belonging to each equivalence class. Being from the same organism, the sequences are nearly identical and can be aligned with a simple tool like ClustalW. To make alignments across species, we download the covariance models from each Rfam family that Rfam maps to PDB chains, then use the Infernal program cmsearch to score each newly released chain against the covariance models to find those that can be confidently mapped (Nawrocki and Eddy 2013, Kalvari *et al.* 2021). The mappings are listed on the Representative Set page at https://rna.bgsu.edu/rna3dhub/nrlist/release/current and on the equivalence class pages available from there. Then, we use the Infernal program cmalign to align all PDB chains mapped to the same Rfam family and store those alignments. We have run several diagnostics, to be published elsewhere, that confirm that the resulting multiple sequence alignments are reliable in conserved regions, both for Watson–Crick paired bases and for bases in hairpin, internal, and multi-helix junction loops. The typical regions that are poorly aligned are where different species have different length helices, where alignment is difficult by nature. Alignments of tRNAs in the Rfam family RF00005 are particularly challenging because there is just one covariance model to cover 20+ tRNAs and all domains plus mitochondrial and chloroplast tRNA. We note here that poor alignments are readily apparent in R3DMCS output because of nonsensical sequence, basepairing, and 3D structure changes. Those are a sign of more significant differences between structures than R3DMCS is designed to investigate.

R3DMCS is built as a Flask web server. Starting from a query set of nucleotides from one RNA 3D structure from PDB, R3DMCS retrieves corresponding nucleotides across the requested scope (EC or Rfam) and at the requested resolution threshold, looks up information for each instance, and geometrically compares instances all against all. R3DMCS builds an output page, described below, to present the corresponding instances and allow interactive investigation of them.

## 3 Input page

The URL of the input page is https://rna.bgsu.edu/correspondence. Users specify the query nucleotides in one of three ways: (i) listing nucleotide numbers individually or in ranges for a given PDB id and chain; (ii) by giving a loop id (from the RNA 3D Hub website) for hairpin, internal, and 3-way junction loops; or (iii) by listing unit ids for individual nucleotides. Format details can be found on the Help page (included as Supplementary Data) or by loading pre-defined examples on the input page. Next, the correspondence scope is chosen, within an equivalence class or across an Rfam family. Finally, one can restrict the query according to a resolution threshold or experimental method.

The input page generates a URL which contains the details of the query, and R3DMCS processes that URL. The URL can be saved to re-generate the query again later. Direct URL input is also available; see the Help page for the syntax. Example queries are available on the input page and throughout the Help page.

## 4 Output page

The R3DMCS output page has several panels. One lists query details and gives summary statistics about the matching instances. Another panel provides a table of retrieved instances, listing the PDB identifier, chain, nucleotide numbers, annotated pairwise interactions and neighboring chains. The heatmap panel provides a visual display of the geometric discrepancy between the retrieved instances. The geometric discrepancy measures structural variation internal to the motif due to the relative location and orientation of bases within the motif (Sarver *et al.* 2008). The instances are ordered by geometric similarity so that instances that are more similar to each other are placed near each other in the heat map and the table. Clusters of similar motifs are easily identified by the dark-colored squares they form on the heatmap. Separate clusters in the heatmap suggest the presence of variable motif geometries that may be correlated with biological function. The coordinate panel displays and superimposes motif instances, with coloring and numbering options and the choice to show or hide residues in the neighborhood of the instance(s). The heatmap, table, and coordinate panels are linked, so that instances can be selected by checking boxes on the table or clicking on the heatmap and then viewed in the coordinate window.

## 5 Example: decoding loop in *Escherichia coli*

**Figure 1.**
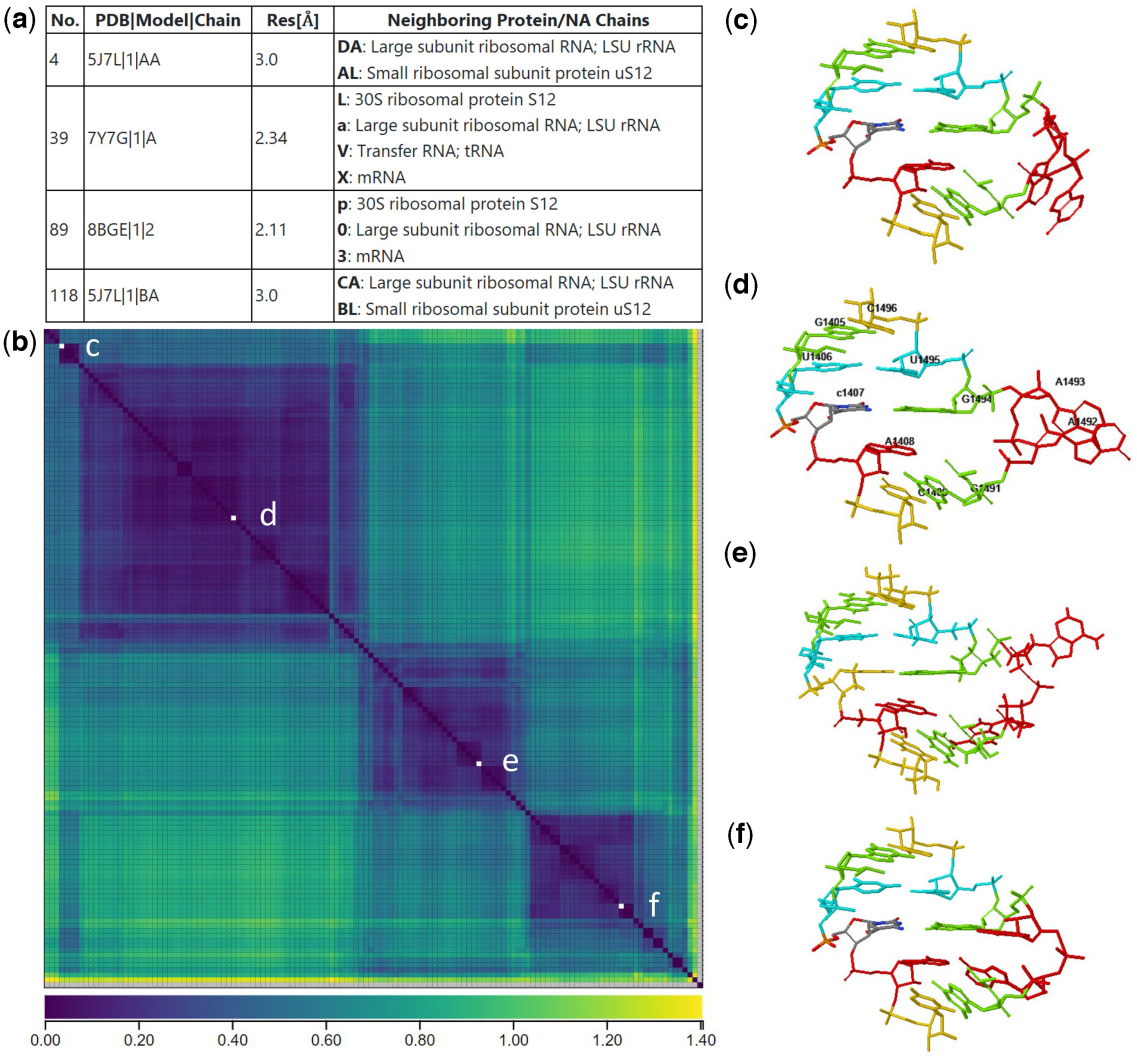
Output page for R3DMCS query of the *E. coli* SSU decoding loop. Four instances are highlighted. (a) Selected cells of the table, listing the PDB id, model, and chain of the four instances, along with the chains within 10 Å of the instance. (b) The heatmap, with the four highlighted instances indicated with white squares on the diagonal. (c) The query instance from 5J7L chain AA, where no tRNA or mRNA are included in the structure. (d) The instance from 7Y7G, with A1492 and A1493 flipped out of the loop due to the presence of tRNA and mRNA (Zhao *et al.* 2023). (e) The instance from 8BGE, with A1493 flipped out but A1492 tucked inside the loop (Jain *et al.* 2023). (f) The instance from 5J7L chain BA, which adopts a different geometry than in chain AA; 5J7L contains two entire ribosomes.

We illustrate the use of R3DMCS to investigate conformational changes with the decoding loop in helix 44 of the *E. coli* small subunit (SSU) ribosomal RNA. The query loop is taken from PDB structure 5J7L, chain AA, residues 1405–1409 on one strand and 1491–1496 on the other (Cocozaki *et al.* 2016). Nucleotides A1492 and A1493 are of particular interest. We set the scope of the query to the *E. coli* SSU equivalence class at 3.0 Å resolution, to see variability across high-resolution structures all from the same organism. We allow all experimental techniques. Click to see the input page with pre-filled fields. As of this writing, R3DMCS maps the query to version NR_3.0_56726.119 of the equivalence class and retrieves 134 instances, but as more structures are solved and added to the *E. coli* SSU equivalence class, R3DMCS will return more instances. R3DMCS finds the neighboring chains, computes all-against-all geometric discrepancy, orders the instances by similarity, and displays the discrepancies in a heatmap. Figure 1 shows selected views of the output page. Several distinct clusters are apparent from the heat map, and one instance is chosen from four of the clusters and displayed, all with the same orientation. Different clusters have different loop geometries, largely corresponding to the presence or absence of other chains needed for translation.

Some additional features of the output page are not shown in Fig. 1. In the table of instances, the nucleotide sequence and number are listed for each retrieved instance, and in later columns all annotated base pairing and stacking interactions between nucleotides in the instance are listed. For example, in the decoding loop, the stacking interaction between A1492 and G1491 is reliably present only in the instances in the cluster marked (f) in Fig. 1. Also on the output page is a panel which summarizes the number of instances each neighboring chain appears in, making it easy to see the full range of interaction partners across different experimental structures.

## 6 Conclusion

The R3DMCS web server provides an automated way to retrieve and compare RNA 3D motifs across structures of the same molecule from the same organism or across species. Doing the same analysis manually would be extremely time consuming because the instances are spread across large numbers of 3D structure files, usually with inconsistent numbering between species. R3DMCS gives a high degree of control over the range of 3D structures to be compared. New structures are added weekly. The output page organizes the instances geometrically and makes it easy to investigate the similarities and differences between instances and their interaction partners. Given the advances in structure determination methods and the rapid rise in the number of structures being solved to atomic resolution, we anticipate the utility of this tool to increase with the deposition of more RNA structures in the 3D structure database in varying conformational states.

## Supplementary Material

btae682_Supplementary_Data

## Data Availability

The data underlying this article are available from the BGSU RNA 3D Hub website at https://rna.bgsu.edu/rna3dhub/.

## References

[btae682-B1] Appasamy SD , Hamdani HY, Ramlan EI et al InterRNA: a database of base interactions in RNA structures. Nucleic Acids Res 2016;44:D266–71. 26553798 10.1093/nar/gkv1186 PMC4702846

[btae682-B2] Chojnowski G , Walen T, Bujnicki JM et al RNA bricks—a database of RNA 3D motifs and their interactions. Nucleic Acids Res 2014;42:D123–31. 24220091 10.1093/nar/gkt1084 PMC3965019

[btae682-B3] Cocozaki AI , Altman RB, Huang J et al Resistance mutations generate divergent antibiotic susceptibility profiles against translation inhibitors. Proc Natl Acad Sci USA 2016;113:8188–93. 27382179 10.1073/pnas.1605127113 PMC4961145

[btae682-B4] Ganser LR , Kelly ML, Herschlag D et al The roles of structural dynamics in the cellular functions of RNAs. Nat Rev Mol Cell Biol 2019;20:474–89. 31182864 10.1038/s41580-019-0136-0 PMC7656661

[btae682-B5] Jain S , Koziej L, Poulis P et al Modulation of translational decoding by m6A modification of mRNA. Nat Commun 2023;14:4784. 37553384 10.1038/s41467-023-40422-7 PMC10409866

[btae682-B6] Kalvari I , Nawrocki EP, Ontiveros-Palacios N et al Rfam 14: expanded coverage of metagenomic, viral and microRNA families. Nucleic Acids Res 2021;49:D192–200. 33211869 10.1093/nar/gkaa1047 PMC7779021

[btae682-B7] Lawson CL , Berman HM, Chen L et al The nucleic acid knowledgebase: a new portal for 3D structural information about nucleic acids. Nucleic Acids Res 2024;52:D245–54. 37953312 10.1093/nar/gkad957 PMC10767938

[btae682-B8] Leontis NB , Zirbel CL. Nonredundant 3D structure datasets for RNA knowledge extraction and benchmarking. In: Leontis N, Westhof E (eds), RNA 3D Structure Analysis and Prediction. Berlin/Heidelberg: Springer, 2012, 281–98.

[btae682-B9] Nawrocki EP , Eddy SR. Infernal 1.1: 100-fold faster RNA homology searches. Bioinformatics 2013;29:2933–5. 24008419 10.1093/bioinformatics/btt509 PMC3810854

[btae682-B10] Parlea LG , Sweeney BA, Hosseini-Asanjan M et al The RNA 3D Motif Atlas: computational methods for extraction, organization and evaluation of RNA motifs. Methods 2016;103:99–119. 27125735 10.1016/j.ymeth.2016.04.025 PMC4921307

[btae682-B11] Petrov AI , Zirbel CL, Leontis NB et al Automated classification of RNA 3D motifs and the RNA 3D Motif Atlas. RNA 2013;19:1327–40. 23970545 10.1261/rna.039438.113 PMC3854523

[btae682-B12] Richardson KE , Kirkpatrick CC, Znosko BM et al RNA CoSSMos 2.0: an improved searchable database of secondary structure motifs in RNA three-dimensional structures. Database 2020;2020:baz153. 31950189 10.1093/database/baz153 PMC6966092

[btae682-B13] Sarver M , Zirbel CL, Stombaugh J et al FR3D: finding local and composite recurrent structural motifs in RNA 3D structures. J Math Biol 2008;56:215–52. 17694311 10.1007/s00285-007-0110-x PMC2837920

[btae682-B14] Zhao X , Ma D, Ishiguro K et al Glycosylated queuosines in tRNAs optimize translational rate and post-embryonic growth. Cell 2023;186:5517–35.e24. 37992713 10.1016/j.cell.2023.10.026

