# Supplementary material for: R3DMCS: a web server for visualizing structural variation in RNA motifs across experimental 3D structures from the same organism or across species
Source: Bioinformatics. 2024 Nov 15;40(11):btae682. doi: 10.1093/bioinformatics/btae682 (PMC11588024; doi:10.1093/bioinformatics/btae682)
Supplement: btae682_Supplementary_Data [file btae682_supplementary_data.pdf]

R3DMCS Help page

Table of Contents

R3DMCS Help page 1
Introduction 2
Input page 2
Step 1 - Select nucleotides 2
RESIDUE NUMBER 3
Individual residues 3
Single range of residues 3
Multiple ranges of residues 3
Step 2 - Correspondence Scope 4
CORRESPONDING NUCLEOTIDES FROM THE SAME MOLECULE IN THE SAME ORGANISM 4
HOMOLOGOUS NUCLEOTIDES FROM THE SAME MOLECULE IN DIFFERENT ORGANISMS 4
Depth 5
Step 3 - Resolution threshold 5
Step 4 - Experimental technique 5
Step 5 - PDB identifiers to exclude 5
Step 6 - Submit 5
URL access 6
Base URL 6
Selection of nucleotides 6
Scope and depth 6
Resolution threshold 7
Experimental technique 7
PDB identifiers to exclude 7
Pre-filled input page 8
Output page and examples 8
Example 1: *E. coli* small decoding loop 8
QUERY INFORMATION PANEL 9
TABLE OF INSTANCES 10
COORDINATE WINDOW 11
HEATMAP 12
NEARBY CHAIN LISTING 16
Example 2: *E. coli* SSU h27 internal loop 17
Example 3: Human LSU H34 hairpin loop 19

Example 4: NMR internal loop 19

Availability ***20***

Limitations ***20***

Internal Server Error 20

Nucleotides must be from the same chain 20

Alignments across different Rfam families are not available 21

Poor alignment quality in some regions of some Rfam alignments 21

Long computation time on large comparisons 21

Partial versus full matches 21

No discrepancy calculated in some cases 21

Introduction

R3DMCS (pronounced like /redmax/) is a web service which maps an input set of RNA nucleotides in one 3D structure from the Protein Data Bank to an output page showing corresponding instances of the motif across 3D structures of the same molecule from the same organism, or across 3D structures of the same molecule from other organisms in the same Rfam family. Target structures can be filtered by resolution, redundancy, and experimental method. The output page lists the corresponding instances in a table as a multiple alignment, with each row listing nearby chains in the structure, basepairs and other pairwise interactions, and structure resolution. The output page shows an all-against-all comparison of the instances of the motif in the form of a heatmap; instances are ordered to put geometrically similar instances near each other. Finally, the output page has a coordinate viewer to visualize one instance at a time, or to superimpose instances for easier comparison.

Input page

This section provides an overview on how to fill up the input page.

Step 1 - Select nucleotides

First specify the nucleotides in one 3D structure that you would like to work with. There are different ways to describe your selection: residue number, loop id, and unit id. A short description for each selection type is given below.

RESIDUE NUMBER

For the most part, each residue in a 3D structure file can be identified by the PDB identifier (like 5J7L), the author-assigned chain identifier (like AA), and residue numbers, also called nucleotide numbers (like 1405 and 1496). Note that the chain identifier is case sensitive, but the PDB identifier is not.

Individual residues

To retrieve the specific nucleotides mentioned above, one would type 1405,1496 in the Selection box, 5J7L in the PDB ID box, and AA in the Chain ID box. The general format for entering individual nucleotide numbers is "**number1,number2,number3**" and repeat for as many individual positions as are needed. Individual residue numbers are separated by commas. Nucleotides 1405 and 1496 are one of the closing basepairs in the decoding loop in the SSU of E. coli. Link to example of individual residue numbers. Note that in that example, some 3D structures model C1496 in syn, creating a separate cluster in the heat map.

Single range of residues

A range of residue numbers can be provided, separating the lower and upper number with a colon character. The format for entering a single range of nucleotide numbers is "**start_position:end_position**". For example, to specify the lower-numbered strand of the decoding loop in the E. coli SSU from 5J7L chain AA, one would type 1405 1409 in the Selection box. Link to example of range of residue numbers.

Multiple ranges of residues

This option consists of entering multiple single ranges of nucleotide numbers separated by commas. This is especially helpful when the motif is an internal loop, a junction loop, or a longrange interaction motif. The general format is "**start1:end1,start2:end2**" and repeat for as many ranges as are needed. For example, to get both strands of the E. coli SSU decoding loop, one would type 1405 1409,1491 1496. Link to example of multiple ranges of residue numbers.

LOOP IDENTIFIER

Each week, the BGSU data processing pipeline extracts hairpin, internal, and junction loops from RNA-containing 3D structure files using the FR3D software. Once the loops are extracted, we label them with unique and stable identifiers. These "loop ids" contain the following three fields, separated by underscores:

- Field 1: Loop type prefix: "HL" for hairpin loops, "IL" for internal loops, "J3" for three-way junctions
- Field 2: PDB ID
- Field 3: A sequentially assigned, three digit number

Users can view the loop_ids for a particular RNA structure by exploring the RNA Structure Atlas pages. See, for example, the loop page for 5TBW, hairpin loop HL_5TBW_007, internal loop IL_5TBW_019, and 3-way junction loop J3_5TBW_003. Loop ids are also used in the RNA 3D Motif Atlas. For example, motif group IL_29549.7 contains 35 instances of the kink-turn internal loop motif. Each of the 35 loop ids there could be used as the starting point to explore variation in the kink turn geometry across different experimental structures.

To specify a R3DMCS query using a loop id, type the loop id in the Selection box, and leave the PDB id and Chain id boxes empty. Link to example of using loop id to specify a query.

UNIT IDENTIFIER

Unit ids allow full ability to specify a particular residue. Unit ids are described on the unit_id page. For example, 5J7L|1|AA|G|1405 and 5J7L|1|AA|C|1496 are the unit ids for one of the flanking pairs in the E. coli SSU decoding loop. Each unit id tells the PDB id, the model number, the chain, the sequence of the residue, and the residue number, and can optionally also tell the insertion code, alternate id, and symmetry operator. Link to example of using unit ids to specify a query.

SPECIAL CASES

In NMR structures, model number is sometimes used. The residue number option above assumes that model 1 is desired, and provides no way to specify a different model. Loop ids can come from models other than 1. Unit ids can be used to specify PDB id, model number, chain id, residue sequence, residue number, alternate id, and symmetry operator. Link to example of using unit ids to specify an NMR model.

Step 2 - Correspondence Scope

Once you specify the nucleotides of interest from a particular structure, R3DMCS can retrieve nucleotides for comparison in one of two ways:

CORRESPONDING NUCLEOTIDES FROM THE SAME MOLECULE IN THE SAME ORGANISM

R3DMCS uses the Equivalence Classes in the BGSU RNA Representative Sets to identify the 3D structures of the same molecule from the same organism. For example, the E. coli SSU equivalence class NR_3.0_56726.129 collects all 166 small subunit ribosomal RNA chains from different 3D structures solved at 3.0Å or better resolution, as of October 15, 2024. Sequence-based alignments between these chains are computed each week for newly added chains, and R3DMCS uses those alignments to find corresponding nucleotides.

HOMOLOGOUS NUCLEOTIDES FROM THE SAME MOLECULE IN DIFFERENT ORGANISMS

R3DMCS uses multiple sequence alignments produced by the BGSU RNA pipeline using Infernal and based on Rfam covariance models, for all PDB chain sequences in each Rfam family. The Rfam covariance models are downloaded with each Rfam release. Infernal and the covariance models are used each week to map PDB chains to Rfam families. For example, the E. coli SSU chain AA from PDB structure 5J7L maps to Rfam family RF00177. To see all of the other chains that map to Rfam family RF00177, one can go to a Representative Set release page and type RF00177 in the Filter box. As of the October 9, 2024 release at 3.0Å resolution threshold, this results in 21 equivalence classes. Of these, 15 correspond to bacterial species, 5 to mitochondrial ribosomes, and 1 to a chloroplast ribosome. The number of chains in each equivalence class varies from 1 to 228. To avoid over-representation from any one equivalence class, the following input parameter can be used when retrieving over different organisms:

Depth

The depth parameter tells the maximum number of chains in each equivalence class to use for the retrieval across different organisms. The chains are prioritized in the order that they are listed on the Representative Set page, which is according to six structure quality factors, which are briefly described on the Representative Set home page.

Step 3 - Resolution threshold

The BGSU RNA site organizes equivalence classes (chains of the same molecule from the same species) by resolution, keeping all structures solved up to a threshold value. Setting the resolution threshold makes it possible to look only at the highest resolution structures. The query will retrieve corresponding motif instances from all chains in the equivalence class with resolution up to the resolution threshold. The default resolution threshold is 3 Ångstroms. Note that NMR structures have no reported resolution, and can be retrieved only by setting the resolution threshold to “all”.

Step 4 - Experimental technique

Choose the desired experimental technique. Users can choose X-ray diffraction, cryo-EM microscopy, NMR, or “all” which includes all techniques above. Example of a loop in an NMR structure. Note that the query above will map to all models in 6MCI and across models in other structures in the equivalence class.

Step 5 - PDB identifiers to exclude

Occasionally a PDB entry will have a very different 3D structure in a specific region, due to a variety of factors such as the presence of a modified nucleotide, a bound ligand, or a modeling feature that sets it far apart from other structures. If it is desired to exclude those instances in order to focus on ones of more direct interest, it is possible to give a list of PDB identifiers to exclude. For example, in the example of individual residue numbers from above, the structures 4V9O and 4V9P model C1496 in syn, which sets them apart from the other instances. Structure 8G2U models the bases in different planes. Also, structure 8GHU has the nonstandard residue ZIV at position 1405, which has two bases in one nucleotide and so is not compared geometrically to the other instances, which is shown with gray cells in the heat map. Listing these three structures under PDB identifiers to exclude removes these instances, and the heat map coloring adjusts accordingly, making it easier to see the slight dissimilarity between the remaining instances.

Step 6 - Submit

After pressing the Submit button, the R3DMCS server will retrieve data for all corresponding nucleotides, calculate all-against-all discrepancies, order the instances by similarity, and render the page in HTML. This process typically takes 5 to 15 seconds, depending on the number of instances returned. However, a larger query or a larger number of instances will take longer; for reference, R3DMCS took almost 223 seconds for a tRNA hairpin retrieval that resulted in 520 instances. See other timing examples below.

URL access

R3DMCS works by responding to a carefully created URL. The only function of the input page described above is to create the URL, and then R3DMCS processes the URL. The URL can be created separately from the input page using the settings described in this section.

Base URL

The base URL is:

- https://rna.bgsu.edu/correspondence/comparison

Selection of nucleotides

It is required to specify a selection, using one of the methods listed above. For example, to specify a hairpin loop in PDB structure 5TBW:

- https://rna.bgsu.edu/correspondence/comparison?selection=HL_5TBW_007

Default settings in the query above are: retrieve within the equivalence class, resolution threshold 3.0Å, all experimental techniques, and no excluded PDB ids.

To use residue numbers, also specify the PDB id and the chain id, and then specify ranges as explained in the examples above. For individual nucleotides:

- https://rna.bgsu.edu/correspondence/comparison?selection=1405,1496&pdb=5J7L&chain=AA&resolution=3.0

For a single range of nucleotides:

- https://rna.bgsu.edu/correspondence/comparison? selection=1405 1409&pdb=5J7L&chain=AA&resolution=3.0

For multiple ranges of nucleotides:

- https://rna.bgsu.edu/correspondence/comparison? selection=1405 1409,1491 1496&pdb=5J7L&chain=AA&resolution=3.0

For multiple ranges of nucleotides plus an individual nucleotide:

- https://rna.bgsu.edu/correspondence/comparison? selection=1405 1409,1491 1496,530&pdb=5J7L&chain=AA&resolution=3.0

Scope and depth

To specify the scope use the “scope” key and values **EC** for **retrievals across the equivalence class of same molecule, same species**, and use **Rfam** for **retrievals across species**.

- https://rna.bgsu.edu/correspondence/comparison?selection=HL_5TBW_007&scope=EC
- https://rna.bgsu.edu/correspondence/comparison?selection=HL_5TBW_007&scope=Rfam

When using scope=Rfam, the **default depth is 1**, but other values can be chosen, so that R3DMCS retrieves additional instances from each species:

- https://rna.bgsu.edu/correspondence/comparison?selection=HL_5TBW_007&scope=Rfam&depth=4

Resolution threshold

To specify the maximum resolution, use the "resolution" key and value chosen from **1.5, 2.0, 2.5, 3.0, 3.5, 4.0, 20.0, all**:

- https://rna.bgsu.edu/correspondence/comparison?selection=HL_4V9F_007&scope=EC&resolution=2.5

Make sure that the query structure is within the resolution threshold.

Experimental technique

To specify the experimental technique, use the exp_method key and values all, x-ray, xray, cryo-em, em, nmr:

- https://rna.bgsu.edu/correspondence/comparison?selection=HL_5TBW_007&scope=EC&resolution=3.0&exp_method=x-ray
- https://rna.bgsu.edu/correspondence/comparison?selection=HL_5TBW_007&scope=EC&resolution=3.0&exp_method=cryo-em
- https://rna.bgsu.edu/correspondence/comparison?selection=IL_2I7Z_001&exp_method=nmr&scope=EC

Note that when exp_method is nmr, then the resolution will be set to all, so that NMR structures will be found and returned.

PDB identifiers to exclude

To exclude specific PDB ids, for example because they have an unusual feature that distracts from the main data analysis, use the exclude key and list PDB ids separated by commas:

- https://rna.bgsu.edu/correspondence/comparison?pdb=5J7L&chain=AA&selection=1405%3A1409&exp_method=all&resolution=3.0&scope= EC&exclude=8GHU,8G2U

In the URL above, we modify the earlier example of nucleotides 1405 to 1409 from chain AA of 5J7L to exclude 8GHU and 8G2U. The instance from 8GHU has the modified nucleotide ZIV in position 1405 and so no discrepancy was calculated. The instance from 8G2U had large discrepancy with nearly every other nucleotide, causing the color range to be mostly blue between all other instances. Excluding those instances makes it possible to better discern the structure of the remaining instances; the maximum discrepancy dropped from 1.26 to 0.39.

Another good example is obtained from the *E. coli* SSU basepair 1405 with 1496; in the query above, two instances have the C modeled in syn, which are real outliers compared to the other instances. Excluding 4V9O and 4V9P together with 8G2U and 8GHU allows us to focus on the other instances.

- https://rna.bgsu.edu/correspondence/comparison?pdb=5J7L&chain=AA&selection=1405%2C1496&exp_method=all&resolution=3.0&scope= EC&exclude=4V9P,4V9O,8G2U,8GHU

Pre-filled input page

To direct the user to the input page with fields already filled in, so the user can modify the fields as desired before requesting the results, use the input_form key with value true:

- https://rna.bgsu.edu/correspondence/comparison?selection=IL_5J7L_014&exp_method=all&resolution=3.0&scope=EC&input_form=True

Note that a significant fraction of the instances on this page have A279 modeled in syn rather than anti in the example above.

Links to pre-filled R3DMCS input pages are provided on all hairpin, internal, and 3-way junction loop pages on the BGSU RNA site; see for example the page for IL_5J7L_014, which is a kink turn.

After loading the input page, it takes 2 seconds for the Submit button to change to blue and become active. That is to slow down bots that might click links to the R3DMCS input page.

Output page and examples

The R3DMCS output page provides query information, a table of instances, a coordinate window, an interactive heat map, and a listing of nearby chains. Each row of the table lists one instance, and shows the PDB id, model number, chain, resolution, nearby chains, nucleotide numbers, and annotated pairwise interactions. The instances are ordered by geometric similarity so that instances that are more similar to each other are placed near one another in the table. The same ordering is used in the heatmap. The heatmap is interactive; clicking the heatmap selects instances, which are then marked in the table and are shown in the coordinate window. These features of the output page are explained in detail in the context of Example 1 below.

Example 1: *E. coli* small decoding loop

Example 1 illustrates the dynamic nature of the decoding loop. During translation, the decoding loop in helix 44 of the small subunit ribosomal RNA makes contact with the mRNA to promote fidelity of translation. The contact is made by two adenine bases, often numbered 1492 and 1493, flipping out of the internal loop. When the mRNA is not present, the adenine bases typically stack inside the internal loop. We can see several different conformations of the internal loop with R3DMCS. We use internal loop IL_5J7L_060 from E. coli as the query. For this illustration, we use resolution threshold 3.0Å and retrieve corresponding instances across the equivalence class of *E. coli* small subunit ribosomal RNA 3D structures.

See the URL to produce the input page for Example 1. The query takes around 30 seconds to return results on 149 corresponding instances.

QUERY INFORMATION PANEL

The upper left panel of the output page, shown below, shows basic information about the query and the corresponding instances. The query nucleotides come from PDB id 5J7L, model 1, chain AA. The standardized name of that chain is the small subunit ribosomal RNA, SSU for short. The query nucleotides are listed; note that residue 1407 is a modified C. Concatenating the PDB|Model|Chain with the query nucleotide sequence and number would give the full unit id, for example, 5J7L|1|AA|G|1405 for the first nucleotide. The Query Organism identifies the species of the PDB chain the query nucleotides are from. Since we chose to retrieve instances from across the equivalence class, the equivalence class identifier NR_3.0_56726.119 is shown. The identifier indicates that the resolution threshold is 3.0Å and that the equivalence class with handle 56726 is on version 119, meaning that since the inception of this equivalence class, the membership has changed 119 times. (R3DMCS always queries the current version of an equivalence class; when this discussion was first written, the version was 119, but by now the version number is well beyond 119.) This query retrieved 134 instances, all of which are from *E. coli* small subunit ribosomal RNA 3D structures. In the all-against-all geometric comparison, the largest geometric discrepancy is 1.40, indicating a moderate level of geometric similarity even between the most dissimilar instances.

Query PDB|Model|Chain

5J7L|1|AA

Query Chain Standardized Name

Small subunit ribosomal RNA; SSU rRNA

Query Nucleotides

G|1405, U|1406, 5MC|1407, A|1408, C|1409, G|1491, A|1492, A|1493, G|1494, U|1495, C|1496

Query Organism

Escherichia coli

Equivalence Class

NR_3.0_56726.119

Number of instances

134

Maximum Geometric Discrepancy

1.40

TABLE OF INSTANCES

The table of instances in the center of the output page lists all 134 instances. In the image below, we show two rows of the table. The query instance is in row 4 and indicates the PDB, model, and chain to be 5J7L, 1, AA. The structure 5J7L was solved at 3.0Å resolution. The columns numbered 1, 2, 3, indicate the query nucleotides, starting with G|1405. The column labeled "Neighboring Protein/NA Chains" indicates chains which have at least one residue within 10Å of one of the nucleotides in the instance on that row. In 5J7L, that includes the LSU rRNA and the SSU protein uS12. The instance in row 52 of the table is from PDB structure 7M5D, solved at 2.8Å. Numbering in *E. coli* 3D structures is quite consistent from one structure to the next, so it is no surprise that the nucleotide numbers in the columns are the same. What differs the most is the nearby chains; in 7M5D three additional chains are nearby, namely Peptide chain release factor RF-1, a tRNA, and an mRNA; their chain identifiers are indicated at the beginning of each line. As we will explain below, the user can visualize residues from these chains in the coordinate window by clicking "Show neighborhood". (Note: over time as more instances are added to the equivalence class, the row in which a specific instance will be listed is likely to change.)

No.	Show	PDB|Model|Chain	Res[Å]	Neighboring Protein/NA Chains	1	2	3	4	5	6	7
1	☐	7M5D|1|2	2.8	**1**: Large subunit ribosomal RNA; LSU rRNA<br>**A**: Peptide chain release factor RF1<br>**q**: Small ribosomal subunit protein uS12<br>**5**: Transfer RNA; tRNA<br>**4**: mRNA	G|1405	U|1406	5MC|1407	A|1408	C|1409	G|1491	A|1492
4	☐	5J7L|1|AA	3.0	**DA**: Large subunit ribosomal RNA; LSU rRNA<br>**AL**: Small ribosomal subunit protein uS12	G|1405	U|1406	5MC|1407	A|1408	C|1409	G|1491	A|1492

The table of instances has these interactivity features: Click on column headers to sort the table rows by the column; click again to reverse the ordering. Use the checkboxes to select instances; selected instances appear in the coordinate window. After clicking one checkbox, shift clicking another checkbox selects all instances between the two, irrespective of the ordering of the rows. Click on the header of the checkbox column to put the checked instances at the top of the table; click again to put them at the bottom. Type text in the Filter box to show only the rows with the text. Click the download button to download the data in CSV format.

COORDINATE WINDOW

Below, we show the instances from 7M5D|1|2 (left) and from 5J7L|1|AA (right), together with their superposition (center). One can use the checkboxes in the table to select which instances to display; when multiple instances are displayed, they are superimposed. The coordinates can be rotated by clicking and dragging in the window. One can turn on or off the display of nucleotide sequence and number. Default coloring uses red for A, yellow for C, green for G, cyan for U, and CPK for modified nucleotides. CPK coloring can be selected for all nucleotides.

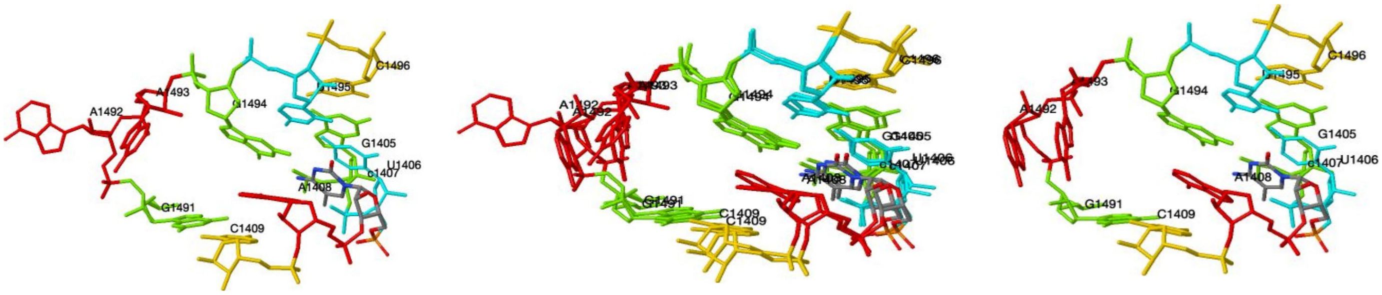

One can click “Show neighborhood” to show all residues within 10 Ångstroms of the selected instance; the instance from 5J7L is shown below. Neighboring nucleotides are shown in gray, and neighboring amino acids are shown in purple.

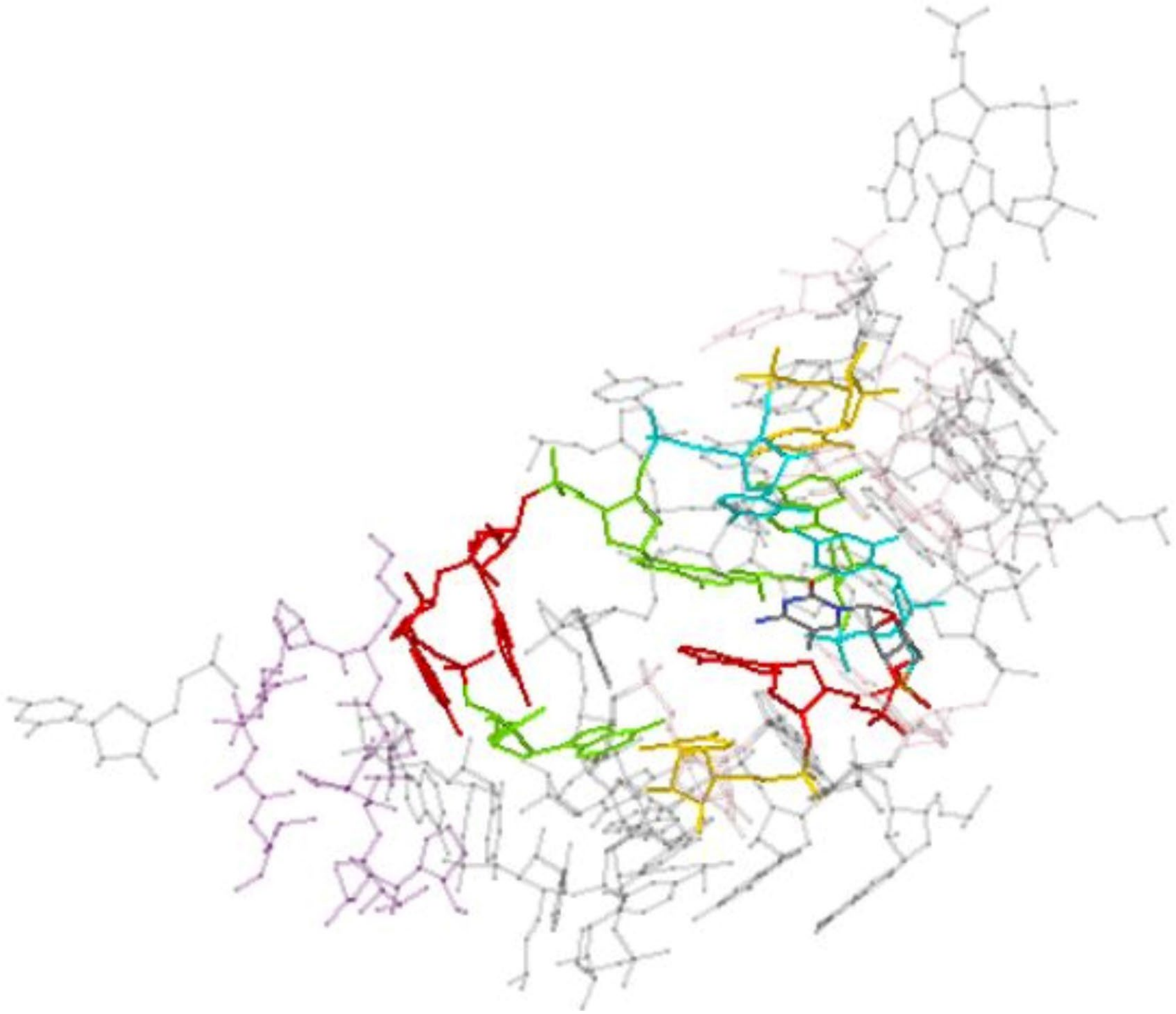

HEATMAP

All instances are compared to one another, all against all, and the geometric discrepancy is calculated. Geometric discrepancy is similar to RMSD, but allows for base substitutions. Geometric discrepancy has two contributions: the location error is the minimum RMSD between glycosidic atoms (N1/N9) when superimposed optimally, and orientation error accounts for different orientations of corresponding bases in the two instances. Units are Ångstroms per nucleotide. See 2008 article on FR3D for more details on the calculation.

If there are N instances, the discrepancies form an N by N matrix, which is displayed as a heat map, with dark colors indicating small discrepancy and light (yellow) colors indicating large discrepancy. In order to make the heatmap easier to understand, the N instances are ordered in such a way that similar instances are put near each other in the ordering. The heuristic for ordering is called tree-penalized path length (tpPL), which is described in detail in a 2023 article on data seriation. With this ordering and the heatmap coloring, clusters of similar instances become apparent, allowing the user to examine a small number of instances from each cluster to understand the variability in the motif.

The heatmap is interactive, in the sense that one can click on the heatmap to select instances, or select instances in the table and see where the instance falls on the heatmap. Briefly,

- Left clicking on the diagonal of the heatmap selects one instance
- Left clicking below the diagonal of the heatmap selects two instances
- Left clicking above the diagonal of the heatmap selects a range of instances
- Right clicking on the heatmap (or control left click) adds to the selection

The selected instance(s) will be indicated by a white square on the diagonal of the heatmap. We illustrate by showing one or more instances from different clusters.

Below, we selected an instance in the center of the large cluster in the upper left of the heat map. The coordinates show A1492 and A1493 flipped out of the internal loop. The table entry shows that the loop nucleotides are close to a tRNA and the mRNA, indicating that this cluster consists of instances reacting to the presence of tRNA and mRNA.

39	☑	7Y7G|1|A	2.34	**L**: 30S ribosomal protein S12<br>**a**: Large subunit ribosomal RNA; LSU rRNA<br>**V**: Transfer RNA; tRNA<br>**X**: mRNA	G|1405	U|1406	5MC|1407	A|1408	C|1409	G|1491

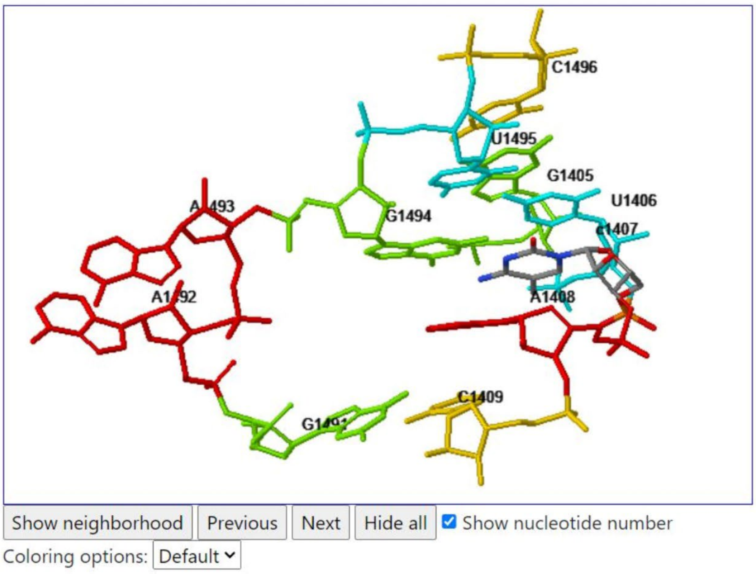

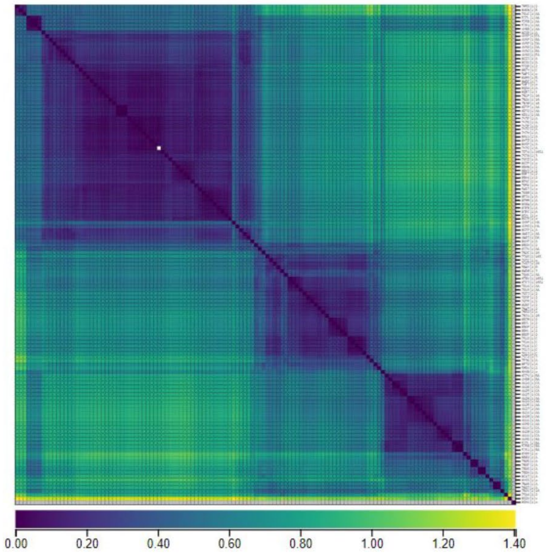

Below, we left clicked above the diagonal to select a range of instances with low mutual discrepancies between them. The superposition of coordinates shows that the instances are all geometrically very similar. The table entries (not shown) indicate that all selected instances are close to tRNA and mRNA.

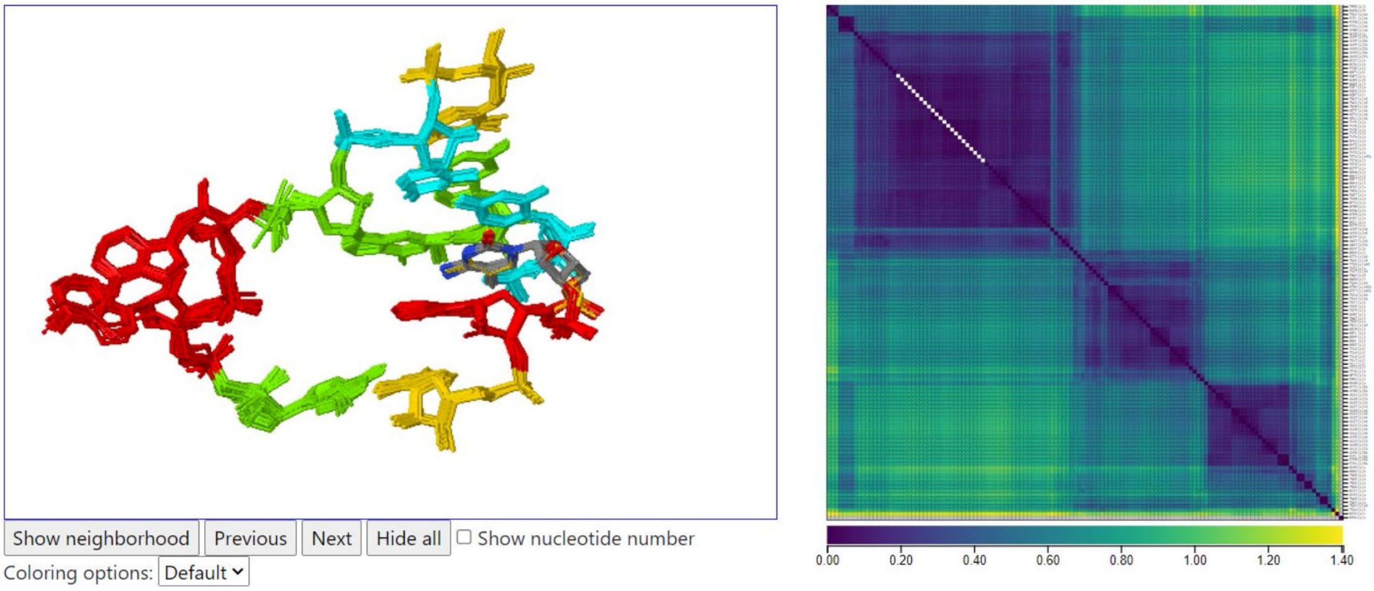

Below, we show the query instance from 5J7L chain AA. It lies in a geometrically distinct cluster, with the A’s not fully extended from the loop. 5J7L does not have tRNA molecules in the structure.

4	☑	5J7L|1|AA	3.0	**DA**: Large subunit ribosomal RNA; LSU rRNA<br>**AL**: Small ribosomal subunit protein uS12	G|1405	U|1406	5MC|1407	A|1408	C|1409	G|1491

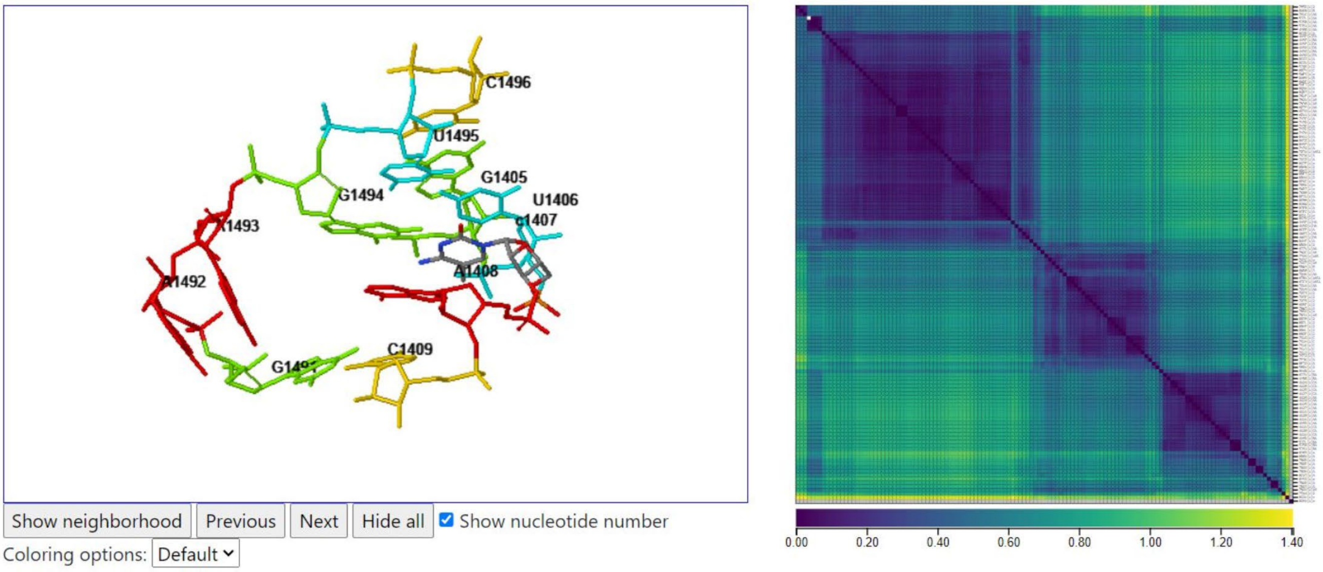

Below, we illustrate how clicking below the diagonal of the heatmap will select two instances, one from the row clicked, one from the column clicked. A1492 and A1493 are in different conformations, but the rest of the internal loop nucleotides maintain much the same geometry between the two instances.

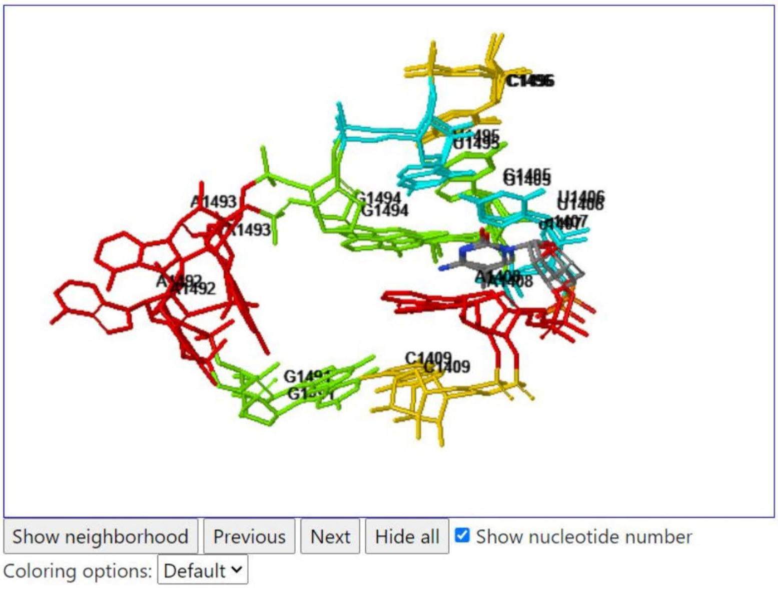

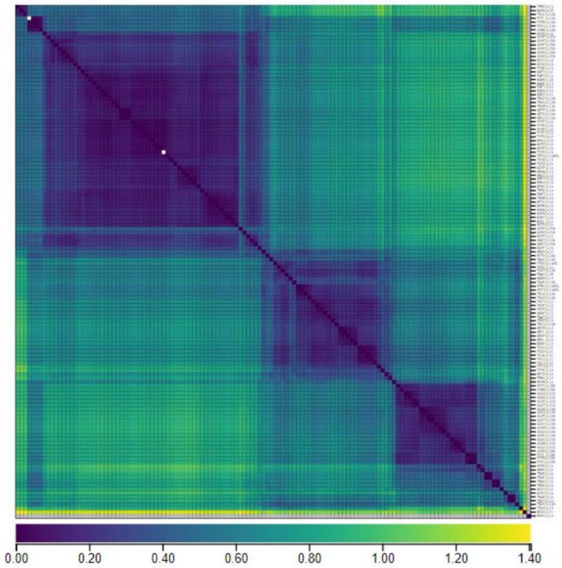

Below, we show an instance from a distinct cluster where A1493 is flipped out but A1492 is stacked inside the internal loop. In this case, no tRNA chain is close to the loop.

89	☑	8BGE|1|2	2.11	**p**: 30S ribosomal protein S12<br>**0**: Large subunit ribosomal RNA; LSU rRNA<br>**3**: mRNA	G|1405	U|1406	C|1407	A|1408	C|1409	G|1491

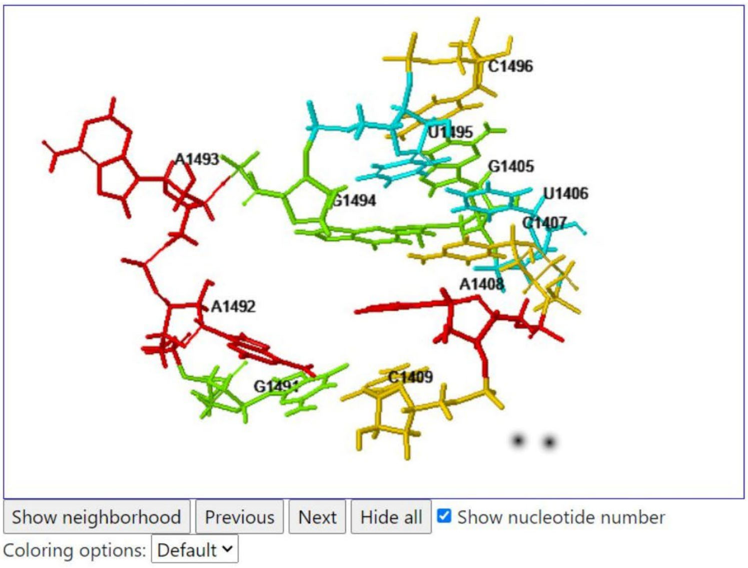

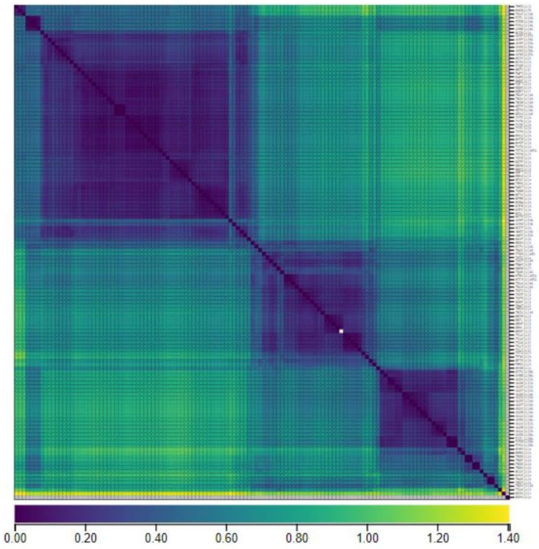

Below, we show one instance from the remaining large cluster of instances. This instance comes from the same PDB id as the query instance, but it comes from chain BA rather than AA. In fact, 5J7L contains two assemblies which are complete ribosomes. Neither have tRNA or mRNA in the structure.

118	☑	5J7L|1|BA	3.0	**CA**: Large subunit ribosomal RNA; LSU rRNA **BL**: Small ribosomal subunit protein uS12	G|1405	U|1406	5MC|1407	A|1408	C|1409	G|1491

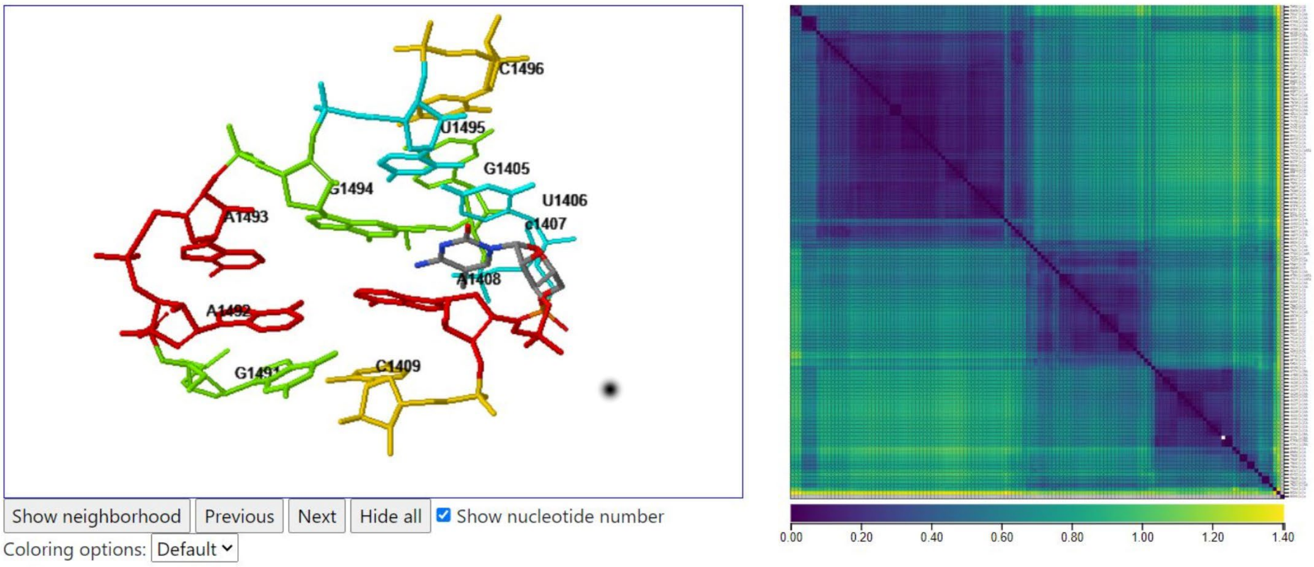

Below, we use the colormap to identify a pair of instances with a large geometric discrepancy between them (yellowish cell in the heatmap) and then click below the diagonal to display them. In one, only A1493 is flipped out, and in the other, only A1492 is flipped out, creating a geometric discrepancy per nucleotide value of 1.1405Å.

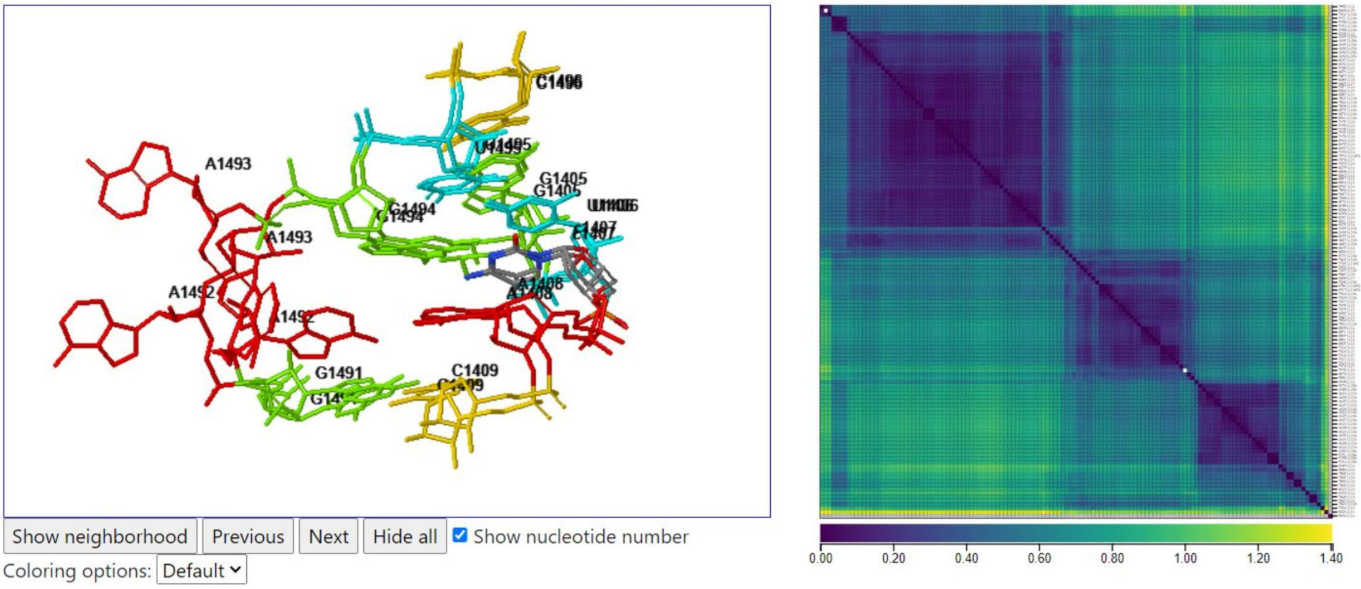

NEARBY CHAIN LISTING

The upper right corner of the output page lists all unique names of nearby chains, with counts of how many times they occur. Below, we show the lines of the table for Example 1.

Unique Neighboring Chains Count

Chain Name	Count
Large subunit ribosomal RNA; LSU rRNA	125
Small ribosomal subunit protein uS12	92
mRNA	70
Transfer RNA; tRNA	70
30S ribosomal protein S12	42
Elongation factor G	12
Viomycin	7
Ribosome hibernation promoting factor	2
Peptide chain release factor RF1	2
Peptide chain release factor RF2	2
Small ribosomal subunit biogenesis GTPase RsgA	2
Capreomycin IA	1
Alternative ribosome-rescue factor A	1
Putative peptide chain release factor homolog	1
Alternative stalled-ribosome rescue factor B	1
Chains: mR	1

Example 2: *E. coli* SSU h27 internal loop

Example 2 studies an internal loop from the small subunit ribosomal RNA helix 27. The core of the loop is the same as the sarcin-ricin internal loop in Helix 95 of the large subunit ribosomal RNA, consisting of a GUA base triple. This recurrent internal loop motif is also called a Gbulge. This example compares instances of the loop across different species whose SSU chains map to Rfam family RF00177. The query loop is IL_5AJ3_023, which comes from chain A of PDB id 5AJ3, which is a small subunit ribosomal RNA from the mitochondrion of *Sus scrofa*. As it happens, there are other 3D structures of the same molecule from the same species, and 5AJ3|1|A is not the representative of the equivalence class of 3D structures, as we illustrate below by showing the table entry in the Representative Set page that contains 5AJ3:

Filter: 5aj3   Showing 1 to 1 of 1 entries (filtered from 2,945 total entries)

#	Equivalence class	Representative	Resolution	Nts	Class members
113	NR_4.0_19551.7 EXACT MATCH Sus scrofa	6GAZ|1|AA (6GAZ) • MITORIBOSOMAL 12S RRNA, MITORIBOSOMAL 12[...] • Electron microscopy • Release Date: 2018-08-08 • Standardized name: SSU rRNA • Source: Mitochondria • Rfam: RF00177	3.1 Å	960	(9) 6GAZ|1|AA, 6GAW|1|AA, 7NQL|1|AA, 7NQH|1|AA, 8OIP|1|AA, 8OIN|1|AA, 5AJ3|1|A, 7NSJ|1|AA, 5AJ4|1|AA

Note that 6GAZ is the representative structure. Note here that the chains in this equivalence class map to Rfam family RF00177, which Rfam labels as being bacterial SSU, but which mitochondrial and chloroplast ribosomes also match well, due to the ribosomes in those organelles originating from bacteria.

Using 5AJ3 as a starting point in the query, R3DMCS maps its 18 nucleotides to other 3D structures which also map to Rfam family RF00177. The query has depth=1, so only one structure, the representative structure, from each equivalence class is returned. Thus, an instance from 6GAZ appears in the output page, not the query from 5AJ3. The query takes about 8 seconds to return results on 27 corresponding instances.

This loop is particularly interesting, because the heat map shows four structures that are quite distinct from the rest, see below where we have selected the instance from 6GAZ and the instance from 5J7L, which is from *E. coli* as in previous examples. The key difference is that the four structures in the lower right of the heat map are all mitochondrial ribosomes, in which position 4 in the sequence is C, whereas the other structures all have G in that position. This example shows that when the G in the base triple in the G-bulge changes to C, the base triple is lost, and the C bulges out of the motif. Apparently, that is not a problem in some mitochondria, but all bacteria and some mitochondria in the 3D structure database have G in that position, and the G participates in the base triple.

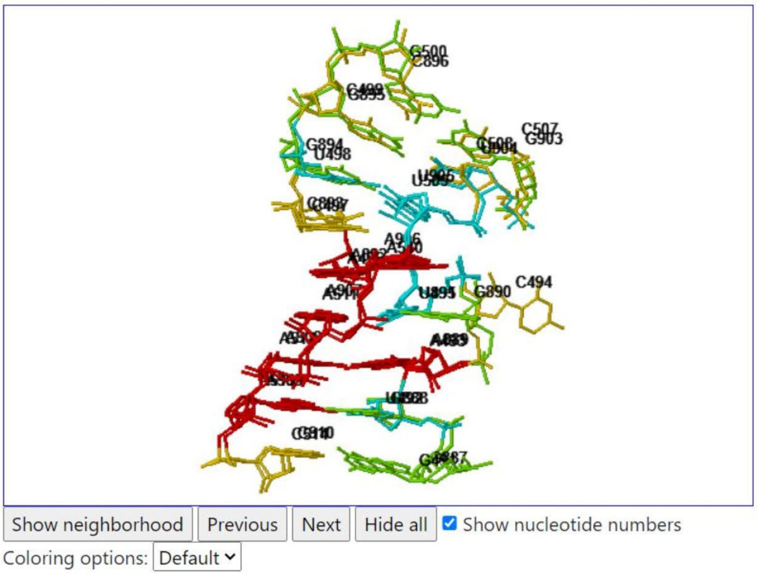

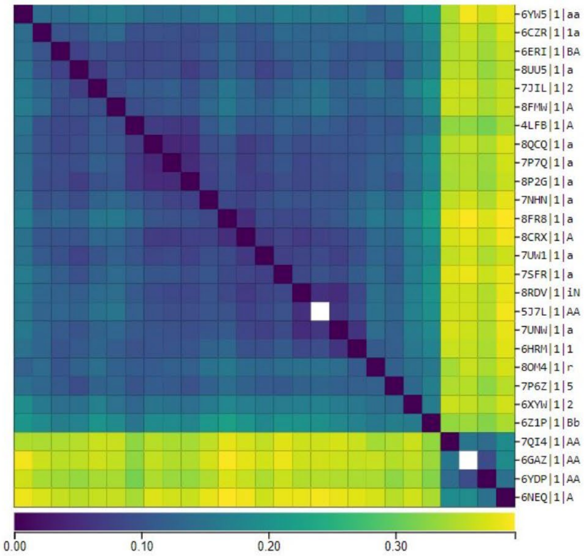

Example 3: Human LSU H34 hairpin loop

Example 3 illustrates the GNRA hairpin loop from Helix 34 of the large subunit ribosomal RNA, compared across different species in the associated Rfam family solved at resolution 4.0A or better, with up to 3 instances from each species. This example illustrates how some structures (in this case, the models of *Triticum aestivum*) model the top adenine base of the GNRA in syn while others model that base in anti. Other variability is also evident. The image below shows the query instance from 8GLP (human) and one instance with the top A modeled in syn; the syn/anti superposition makes a characteristically symmetric image which can be spotted relatively easily. Modeling differences such as this often explain the difference between clusters of instances. This query takes about 15 seconds to return results on 58 corresponding instances.

54	☑	8IPB|1|RB	3.40	**pa**: 40S ribosomal protein uS15<br>**ib**: 40S ribosomal protein uS17<br>**GB**: 60S ribosomal protein eL43<br>**aa**: Small subunit ribosomal RNA; SSU rRNA	*Triticum aestivum*	*Eukarya*	NR 4.0 66212.2	G|847	G|848	A|849	A|850

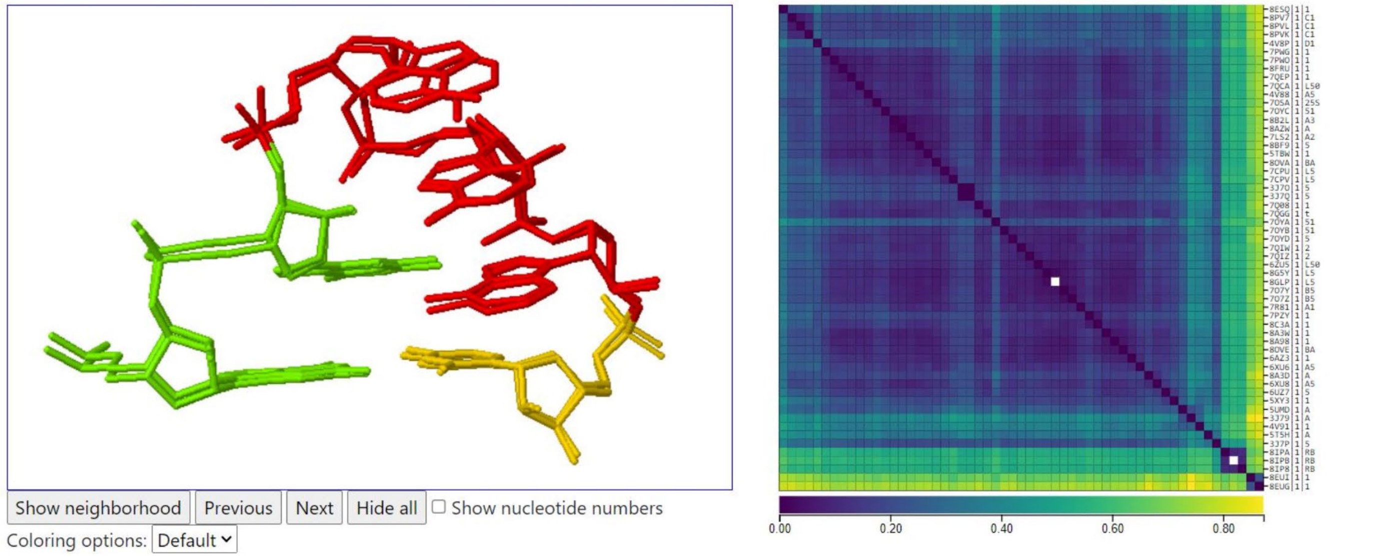

Example 4: NMR internal loop

Example 4 illustrates the variability of an internal loop across 10 models in each of two 3D structures of 7SK RNA solved by solution NMR. The selected instances show four different possible orientations of U41. When starting with an NMR structure, use experimental technique ‘‘NMR’’ or use experimental technique ‘‘all’’ and resolution threshold ‘‘all’’. One can use R3DMCS to view the variability across models for a single PDB entry solved by NMR, even if the equivalence class has only one structure in it. This query takes about 3 seconds to return 20 instances.

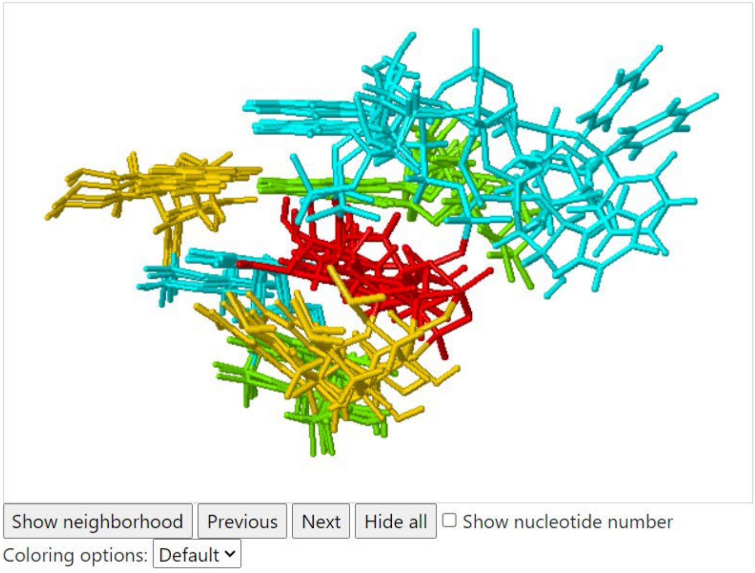

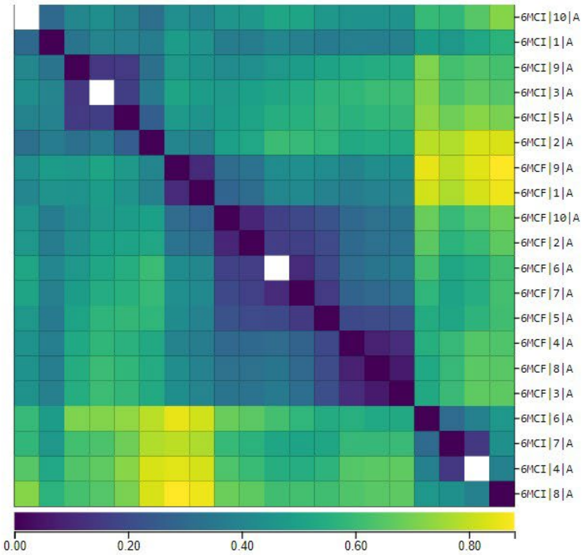

Availability

R3DMCS is freely available to non-commercial users. Users need not register in order to use the service and there is no login requirement.

The code behind the web server is posted on GitHub at https://github.com/BGSU-RNA/RNA3D-correspondence

Limitations

In this section we document known limitations. We hope to remove some or all of these limitations in the future.

Internal Server Error

We have worked hard to identify and eliminate situations in which the page does not return results. Some problems occur intermittently, leading to the message Internal Server Error. When a browser tab with R3DMCS results has been open long enough that the browser identifies it as a stale tab, switching to that tab may result in an Internal Server Error. Reloading the page will often work. We will continue to try to track down and fix errors.

Nucleotides must be from the same chain

The nucleotides in a query must be from the same chain. For example, some internal loops in the eukaryotic ribosomal large subunit (LSU) have one strand in the 5.8S rRNA and one in the long LSU chain, see for example the large internal loop IL_8GLP_021 from Homo sapiens. In principle, it would be possible to retrieve aligned nucleotides across multiple chains, but in practice there are many edge cases that are difficult to cover.

Alignments across different Rfam families are not available

With the ribosomal small subunit (SSU) and large subunit (LSU), Rfam provides separate families for archaea, bacteria, and eukarya. R3DMCS can retrieve and compare motifs within each family, but at the moment does not provide alignments across those different domains. We will remove this limitation when we can provide sufficiently accurate cross-domain alignments. In the meantime, you can produce text-based alignments using the map_across_chains tool on our API page.

Poor alignment quality in some regions of some Rfam alignments

The alignments produced across PDB chains in an Rfam family are sometimes inaccurate, especially in regions where the secondary structure is variable between organisms. This will generally show itself with two or more clearly separated clusters in the heat map, and visual inspection will show that the sets of nucleotides in the two sets bear no resemblance to each other. tRNA alignments are particularly susceptible to this problem, partly because Rfam has a single family for all tRNAs from all domains, and they don't all align perfectly well. Alignment in variable regions is difficult, and perhaps not meaningful because different species simply have different 3D structures. R3DMCS can make it clear that the 2D or 3D structures differ enough in that region to require further study.

Long computation time on large comparisons

R3DMCS can retrieve hundreds of instances, but the all-against-all geometric comparison scales as the square of the number of instances, which can take a few minutes in some cases. The amount of time it took to create the output is shown on the bottom of the output page. It is a good idea to start with a strict resolution threshold or with a low equivalence class depth at first. For reference, R3DMCS produced results for a 92-nucleotide query with 149 matching instances in 87 seconds. R3DMCS is primarily designed for motifs up to about 30 nucleotides.

Partial versus full matches

In some cases, only some of the query nucleotides can be aligned to nucleotides in another structure, which is called a partial alignment. Partial alignments are not returned by R3DMCS. In some cases, only partial alignments are found, and R3DMCS reports that the query returned no results. If you are interested in partial matches, or want to understand why R3DMCS gives no results, see the map_across_chains route on our API page.

No discrepancy calculated in some cases

Some 3D structures have nucleotides with missing atoms, for example, missing base atoms. Others have modified residues that have not been fully processed by our pipeline. These situations can explain cases where no discrepancy is calculated between some instances, even though the nucleotides are shown in the table and in the heat map.
